# Do Bells Affect Behaviour and Heart Rate Variability in Grazing Dairy Cows?

**DOI:** 10.1371/journal.pone.0131632

**Published:** 2015-06-25

**Authors:** Julia Johns, Antonia Patt, Edna Hillmann

**Affiliations:** ETH Zurich, Institute of Agricultural Sciences, Animal Behaviour, Health and Welfare Unit, Zurich, Switzerland; Queen Mary, University of London, UNITED KINGDOM

## Abstract

In alpine regions cows are often equipped with bells. The present study investigated the impact of wearing a bell on behaviour and heart rate variability in dairy cows. Nineteen non-lactating Brown-Swiss cows with bell experience were assigned to three different treatments. For 3 days each, cows were equipped with no bell (control), with a bell with inactivated clapper (silent bell) or with a functional bell (functional bell). The bells weighed 5.5 kg and had frequencies between 532 Hz and 2.8 kHz and amplitudes between 90 and 113 dB at a distance of 20 cm. Data were collected on either the first and third or on all 3 days of each treatment. Whereas duration of rumination was reduced with a functional bell and a silent bell compared with no bell, feeding duration was reduced with a silent bell and was intermediate with a functional bell. Head movements were reduced when wearing a silent bell compared with no bell and tended to be reduced when wearing a functional compared to no bell. With a functional bell, lying duration was reduced by almost 4 hours on the third day of treatment compared with the first day with a functional bell and compared with no bell or a silent bell. All additional behavioural measures are consistent with the hypothesis of a restriction in the behaviour of the cows wearing bells, although this pattern did not reach significance. There was no treatment effect on heart rate variability, suggesting that the bells did not affect vago-sympathetic balance. An effect of experimental day was found for only 1 out of 10 behavioural parameters, as shown by a decrease in lying with a functional bell on day 3. The results indicate behavioural changes in the cows wearing a bell over 3 days, without indication of habituation to the bell. Altogether, the behavioural changes suggest that the behaviour of the cows was disturbed by wearing a bell. If long-lasting, these effects may have implications for animal welfare.

## Introduction

Noise is described as an acute, chronic or intermittent sound [1], which can act as a potential stressor in farmed species such as pigs [2], [3], horses [4], goats [5],[6] and cattle [1],[7], [8], [9]. In piglets, Talling and colleagues [1] found that, within 15 min of acute exposures to various noise stimuli at 80–97 dB once per week over a total of 4 weeks, initial increase in heart rate and locomotion indicated an activation of the pigs’ defence mechanisms, followed by habituation. In another study, exposure to repeated noise during daily sessions of 2 hours over a period of 4 weeks caused changes in neuroendocrine regulations that reflected a state of chronic stress in growing pigs [10]. Horses [4] and goats [5] spent less time feeding when exposed to acute noise for 1–2 min in a test arena. The goats habituated to the noise (chime of bell and sinusoidal sound with maximum amplitude of 96 dB) within 3 days, i.e. from the third day of playbacks there was no difference between baseline and playback values in feeding and vigilance behaviour [5]. Whereas these studies were conducted in rather artificial situations, farm animals are confronted with several acoustic stimuli throughout their lives, including ventilation, feed carts, barn cleaners or the vacuum machine in the milking parlour. The noise of the latter is considered to be stressful for dairy cows as it resulted in both fearful reactions and avoidance in a y-maze choice task [9]. When exposed to playback of noise recorded in milking parlours and played at 85 dB in a raceway 3 times daily, heifers showed an increased heart rate on day 1 and faster transit times on days 1–4, indicating an escape reaction, but habituated over a 5 day period with daily exposure [8]. A reduction in noise and vibration in the milking parlour for 3 months improved udder health, which was, however, mediated mainly by the reduction in vibration rather than noise [11]. Furthermore, exposure to aircraft noise in the waiting area just before milking did not lead to behavioural reactions in dairy cows [1]. When examining the effect of noise on cow behaviour, noise exposure was conducted either in a raceway or in the waiting area just before or during milking, i.e. at times when cows were already aroused, and the effects of noise during resting times were not taken into account [1], [8], [9], [11].

In alpine regions cows are often equipped with a bell throughout the summer season, i.e. for several months, to ensure that farmers can locate their animals on the wide alpine pastures, many areas of which are obstructed from the view. Various types of bells are available, ranging from small ones for goats and calves to large and heavy ones that are used for traditional purposes like seasonal cattle drives (‘Alpabzug’) or exhibitions. Whereas cows are equipped with the larger and heavier bells during these traditional purposes, the smaller bells are used on pasture. However, although the bells used on pasture are smaller than the ones used during traditional purposes, the animals are exposed to them continuously throughout the day for several months. Consequently, cows are also exposed to the bells during resting periods. The chime of a bell is characterised by high and varying amplitudes, varying frequencies and sounds arising intermittently, depending on the movement of the bell. These characteristics have been shown to be more aversive than uniform sounds of similar amplitudes in pigs [2], [12], goats [5] and lambs [13]. As cows have a well-developed hearing capacity [14], a bell may thus lead to deviations in behaviour, indicating aversiveness, and may lead to reduced welfare in the long term if the animals do not habituate to the sound of the bell.

In ruminants, feeding and rumination are indispensable activities and provide useful information regarding health and welfare [15], [16]. Rumination in dairy cattle is associated with saliva production, which helps buffer the acidic conditions in the rumen and prevent rumen acidosis [17], [18]. A reduction in rumination time might result in a reduction of saliva production and eventually challenge health through an increased risk of rumen acidosis.

Although rumination can take place during standing, it mainly takes place while cows are lying [18]. As feeding and lying are mutually exclusive behaviours, there is direct competition for the time allocated to each of them. The time cows spend lying down is an important measure of cow comfort, and cattle welfare standards are increasingly addressing the issue of lying times in dairy cattle [19–22]. The need for lying is described as relatively constant (13h per day [23]) and can dominate other basic needs after only a few hours of forced standing [24], [25]. A lying duration of 11–13h per day is widely recommended as best practice, and reduced lying times are a risk for lameness, especially in cows kept indoors [26–29]. In addition to behaviour, changes in the vago-sympathetic balance (reflected by heart rate variability parameters) have been used as an animal welfare indicator which allows comparing different management procedures, technologies and handling methods in terms of animal welfare [30], [31], [32].

To our knowledge, the impact of wearing a bell on welfare-related indicators has not been investigated in dairy cows. We examined whether and how wearing a functional bell (exposure to weight and chime) and a bell that does not produce any noise (exposure to weight only) for 3 days affects behaviour as well as vagal activity of cows compared with not wearing a bell. Bells become audible when the animals move their heads. Thus, we were interested in whether the cows would move less to quieten the bell or to possibly reduce the burden of its weight. As a consequence, we expected feeding and ruminating durations to decrease because both behaviours include head movements. Moreover, we expected lying duration to increase and locomotion activity as well as head movements to decrease as an adaptation to wearing a functional bell. In addition, herd members may increase the distance to a cow that is wearing a functional bell to avoid the sound. Furthermore, wearing a bell may lead to a change in vago-sympathetic balance resulting in reduced heart rate variability. All these changes were expected to be more pronounced when cows were exposed to both the chime and the weight of the bell compared with only the weight. According to the finding that goats were habituating to once daily minute-long playbacks of bell sounds within 3 days [5], we expected cows to habituate to the bells within the 3 day observation time and, thus, measurements to return to baseline values by the third day.

### Animals, materials & methods

To test our hypotheses, dairy cows were equipped with no bell (control), with a bell with inactivated clapper (silent bell) or with a functional bell (functional bell) for 3 days each in a balanced order. During the treatment period, lying, feeding and ruminating behaviours, leg and head movements, nearest neighbour distances and heart rate variability were recorded. The study was performed between June and November 2012 on a working farm near Zurich, Switzerland (47°31′05.48″ N; 8°50′13.15″ E; 429 m.a.s.l.). The owner of the land gave permission to conduct the study on this site. Procedures used on the animals included fixing a belt for heart rate measurements, a halter and a pedometer. All cows used in this study had previously been habituated to wearing these devices and were used to human contact. Hence, no negative effect of either the equipment or the handling on the cows’ behaviour was expected. The study did not involve endangered or protected species. Ethical approval to conduct the study was obtained from the Zurich Cantonal Veterinary Office, Switzerland (Approval No. 77/2012). Animal care and all experimental procedures were in accordance with the ARRIVE guidelines for animal research [33].

### Animals, housing and management

In this study, we used 19 dry, multiparous Brown-Swiss cows with an average milk yield of 8,000 kg per 305 days of standard lactation, between 3 and 10 years of age. All cows had previous experience with wearing a bell, as all of them had been equipped with a bell on an Alp for 4–5 months when they were 1 year old. Because the experiment had to be conducted on pasture, its duration was limited to the grazing period (end of May–mid-November). During the experiment, the cows were managed under standard summer grazing conditions with 24 hours access to two flat lowland pastures of approximately 3.5 ha in total. Trees provided protection against rain, wind and sun. Hay was provided ad libitum in a round rack with headlocks, and the cows had access to a water trough 24 hours a day. The herd of dry cows was managed in that cows were added to the herd approximately 5 weeks before calving and were removed from the herd 3–5 days before the estimated calving date. Thus, the herd varied in size and consisted of both focal and non-focal cows (Table 1). During the experiment, the average temperature and precipitation were 15°C (min/max: 0/23.5°C) and 13 mm (min/max: 0/485 mm), respectively.

**Table 1:** Experimental design.

Batch	Number of focal cows	Treatment order			Total number of cows on pasture
		1	2	3	
June 20 to July 5	5	No bell	Functional bell	Silent bell	7–9
July 11 to July 26	3	Functional bell	Silent bell	No bell	5–7
July 31 to August 15	3	No bell	Silent bell	Functional bell	4–6
August 29 to September 14	2	Functional bell	No bell	Silent bell	4–6
September 25 to October 10	3	Silent bell	No bell	Functional bell	6–9
October 14 to November 8	3	Silent bell	Functional bell	No bell	9–10

Calendar date, number of animals and order of treatments per batch are shown as well as total number of animals present on pasture during each experimental period.

### Data collection

**Fig 1:**
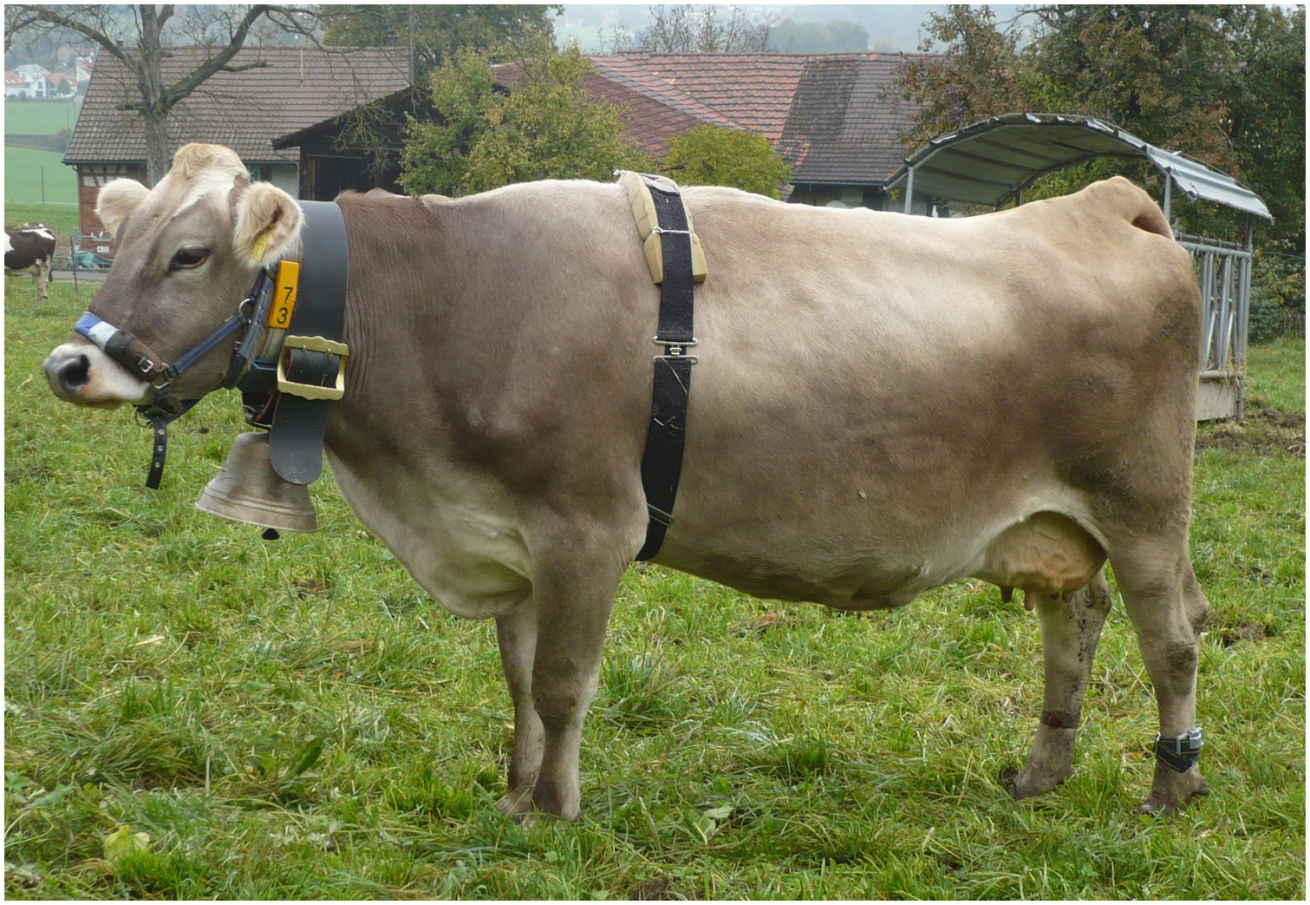
Experimental cow wearing a bell and measuring equipment. A thorax belt for measuring heart rate variability, a halter for recording feeding, ruminating, and head movements, and a 3D accelerometer at the hind leg for recording activity and lying were used for data recording after 24 hours of habituation.

The experiment was conducted during the grazing season (May to November) in six consecutive batches using one unique group of focal cows per batch. Each group of focal cows consisted of two to five individuals with groups being tested one after the other (Table 1). The number of animals per group and batch depended on the number of dry cows available. Each focal cow was exposed to three different treatments: control treatment (‘no bell’), a bell with inactivated clapper (‘silent bell’) or a functional bell (‘functional bell’). For each batch, the order of treatments was balanced over groups and each treatment lasted for three consecutive days. We chose this duration for two reasons: first, farmers said that cows would get used to bells very quickly (within hours). Second, we found in a previous experiment that goats habituated to a playback of a bell within 3 days when exposed to the sound for only 1 min daily [5]. We thus assumed that cows would begin to habituate to a bell they wear 24 hours daily within 3 days, eliminating the need for measurements beyond 3 days. To minimise the risk of carry-over effects, we allowed a break of 3–4 days between two treatments. Due to the memory capacity of the data loggers, data were collected on the first and third experimental day of each treatment for lying behaviour and activity (hind leg acceleration). Nearest neighbour distance and heart rate variability were also recorded on days 1 and 3, whereas feeding and rumination behaviours as well as head movements were recorded on all 3 days of each treatment. This resulted in 19 cows tested in 3 treatments, each treatment lasting 3 days with a break of 3–4 days in between. Depending on the variable, measurements were taken on either 2 or 3 days. In the morning (between 0800 h and 0900 h) of the day before the start of the first treatment, cows were fitted with the measuring equipment (halter, logger with 3D-accelerometer and thorax belt, Fig 1) for habituation (i.e. a habituation period of 24 hours before measurements started) and were then wearing the devices continuously until the first treatment was finished.

In the morning (between 0800 h and 0900 h) of the first day of the second and third treatments, the focal cows were again fitted with a halter, a 3D-accelerometer logger on the hind leg and a thorax belt. At this time on the first day of each bell treatment, cows were also fitted with a silent or a functioning bell (Fig 1, S1 Video). During the fitting procedure, cows were restrained in the headlocks of the hay rack on pasture (S1 Fig). Then, the measuring devices were attached in the following order: 1. halter, 2. accelerometer on the leg, 3. belt for heart rate variability measurements, 4. bell. Attaching the devices took approx. 5 min per cow, and the cows usually did not show signs of disturbance except one cow that tried to get rid of the bell by vigorously shaking its head, jumping and running. We had to exclude this cow from the experiment to avoid severe stress and/or injuries to the animal. All handling procedures and observations were conducted by the same person (Julia Johns). Data collection started when all focal cows were fitted with the measuring equipment and bells and released from the hay rack. After the first 24 hours of data collection (first experimental day), the loggers on the leg and the thorax belts were removed and data were downloaded. In the morning of the third day (third experimental day), cows were again equipped with the loggers on the leg and the thorax belts and data collection started for another 24 hours. The halters were not removed and data collection lasted continuously over the 3 days of each treatment. The data from the loggers of the halters were downloaded after the third experimental day. Based on this experimental design, only the focal cows were observed, except for recording the distance to the nearest neighbour, for which all cows on pasture were included. The cows of the same experimental group (six groups) were observed on the same calendar dates. Cows that had finished the treatments stayed in the herd until calving but were not observed anymore. Thus, herd size varied in the course of the experiment (Table 1).

### Bells

**Fig 2:**
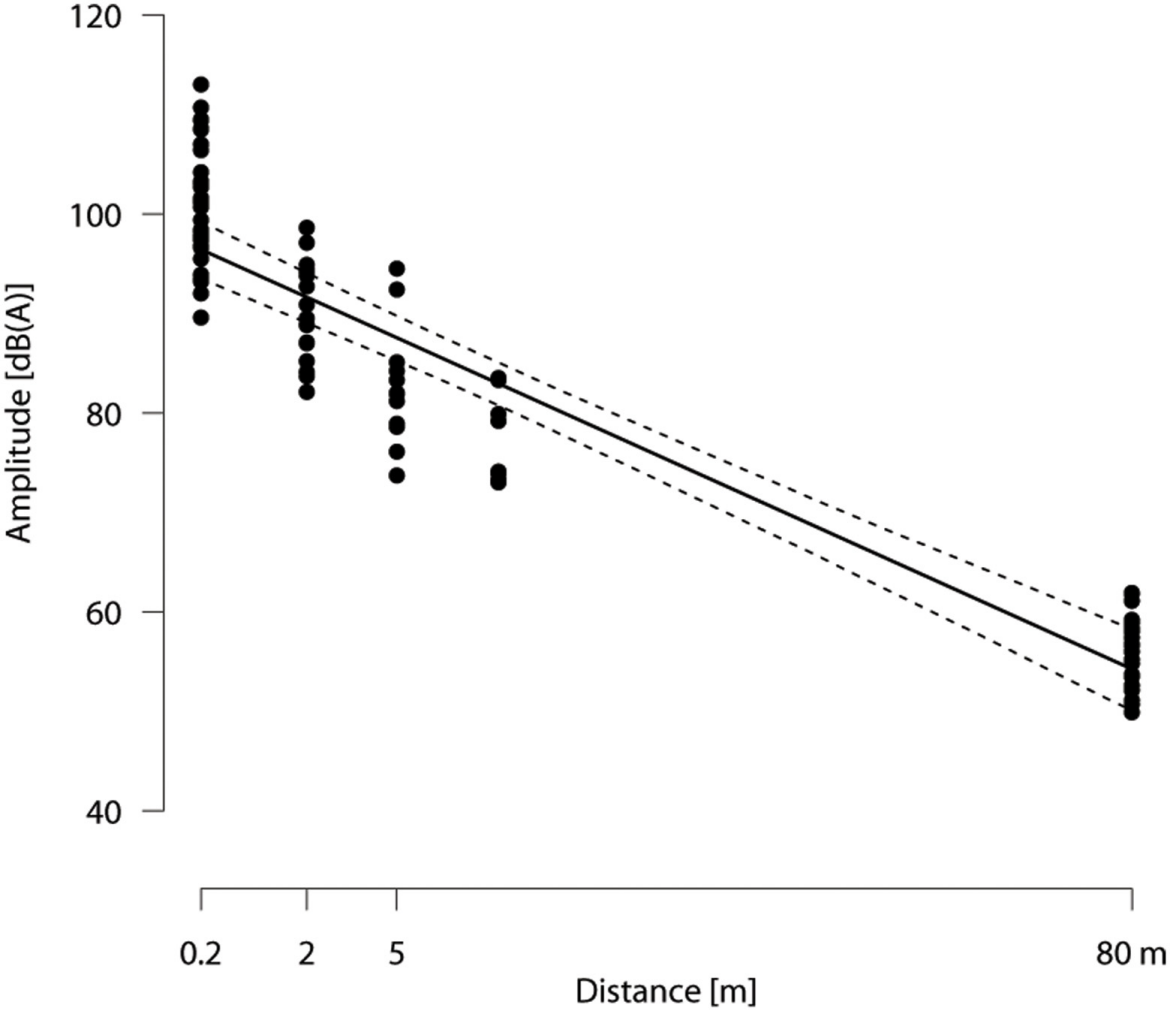
Amplitudes of the bells at distances up to 80 m [dB(A)]. Solid line: model estimate, dashed lines: 95% intervals of confidence based on a model predicting amplitude by the square root of the distance, controlling for bell identity and multiple measurements per distance.

The casted cow bells that were used in this study were bells made from a bronze containing about 23% tin known as bell metal, and all were similar in size and identical in weight (5.5 kg, approx. 1% of cow body weight) including the strap used to attach the bell to the neck of the cows (Fig 1). The bells were part of a traditional set of bells for cows that would produce a harmonised sound when being worn by cows. The sound of the bells was measured at a distance of 20 cm, which corresponds to the estimated distance between the bell and the cow’s ears (S1 Sound File). It was characterised by a first frequency band of 532–875 Hz (the lowest visible band on the spectrogram) and a peak frequency of 1.2–2.8 kHz (frequency of highest amplitude, as measured on a spectrum; details are shown in Table 2). In addition, the amplitudes of the bells were measured at distances up to 80 m to see the distance at which the amplitude of the bells decreases substantially (Fig 2). The measurements were conducted by two persons, one moving the bell and the other recording sound and amplitudes. The bells were moved in a standardized way, aimed at mimicking a walking cow.

**Table 2:** Frequency characteristics and amplitudes of the bells used in the experiment recorded at a distance of approx. 20 cm while manually moving the bells in a standardized way for 1 min each.

Bell	First frequency band (Hz)	Peak frequency (kHz)	Mean amplitude (dB[A]) and range (min–max)
1	609	2.6	104.7 (98–113)
2	581	2.8	99.6 (93–109)
3	581	1.7	96.7 (90–104)
4	875	1.2	106.4 (101–111)
5	532	1.3	97.6 (93–103)

Sound analysis was done using PRAAT software; amplitude analysis was done using SoundTest-Master, Laserliner, Umarex GmbH & Co. KG, Arnsberg, Germany.

Amplitudes were measured in dB(A) by using a precision noise level measuring instrument with integrated long-term storage (SoundTest-Master, Laserliner, Umarex GmbH & Co. KG, Arnsberg, Germany). The A-weighting scale assigns low weights to the low-frequency tones, to which the human ear and the ears of some animals are less sensitive, and high weights to the typically more audible high-frequency tones [34]. Thus, the tones were adjusted to match the cows’ (potential) hearing capacity. For the sound analysis, PRAAT v.5.3.41 DSP Package was used.

### Behavioural observations

The experimental design resulted in a sample size of 38 cow-days (i.e. 19 cows × 2 days) per treatment for lying behaviour, leg accelerations and nearest neighbour distance, and 57 cow-days (i.e. 19 cows × 3 days) per treatment for feeding and rumination behaviours and acceleration of head movements. Due to technical problems, data collected on five animals of the treatment ‘functional bell’ and on one animal of the treatment ‘silent bell’ could not be used for the analysis of lying behaviour and activity, and data collected on six animals of the treatment ‘functional bell’ and on five animals of the treatments ‘no bell’ and ‘silent bell’ could not be used for feeding and rumination analysis. One cow approached calving and had to be excluded from the treatment ‘functional bell’, and data collected on another cow had to be excluded from the treatment ‘silent bell’ because the strap of the bell was broken. Concerning lying behaviour and leg acceleration, this resulted in a remaining sample size of 34 cow-days for the treatment ‘functional bell’, 38 cow-days for ‘silent bell’ and 38 cow-days for ‘no bell’. For feeding and rumination behaviours, we had a remaining sample size of 46 cow-days for ‘functional bell’, 47 cow-days for ‘silent bell’ and 52 cow-days for ‘no bell’.

#### Lying behaviour and leg movements

According to the method described by Helmreich and colleagues [35] as well as Patt and colleagues [36], [37], lying behaviour and activity during locomotion were recorded using a commercial 3D-acceleration logger attached to the left hind leg of each cow (MSR145, MSR Electronics GmbH, Seuzach, Switzerland; 18 × 14 × 62 mm, 33 g). Acceleration in the direction of the y-axis, parallel to the longitudinal axis of the cow’s hind leg, was recorded. Sampling rate was set at 10 Hz (10 measurements/s) and sensitivity of the sensor at ± 10 *g*. The accelerometer was powered by a 260 mAh lithium-polymer battery, rechargeable via USB connection, which enabled measurements for several days. Data were recorded on an integrated memory chip with a capacity of 2 million data points, transferred to a computer via USB connection and stored in a Microsoft CSV-file. MSR PC-Software was used for data transfer and analysis as well as settings of the data loggers [38]. Due to the way the logger was attached to the cow’s left hind leg, acceleration values observed while the cow was lying (quietly) and standing equalled 0 *g* and −1 *g*, respectively. When the leg was moving, acceleration values reached values both higher and lower than −1 *g*. The different positions of the cows’ hind legs while lying down (almost horizontal) as opposed to standing and walking (almost vertical) meant that the lying duration per cow could be calculated per 24 hour period. Leg movements were calculated during the periods in which the cows were standing or walking, i.e. not lying, and the sum of acceleration changes per 24 hours was calculated in *g*.

#### Feeding and rumination behaviours, and head movements

According to the method described by Nydegger and colleagues [39] as well as Braun and colleagues [40], feeding and rumination behaviours of the focal cows were recorded using a halter (500 g) that included a commercial logger (MSR145, MSR Electronics GmbH; 31 × 31 × 72 mm, 33 g) fitted with a pressure-sensitive sensor combined with an oil-filled silicon tube (40 g). This tube was attached to the halter above the cow’s nose. Opening of the mouth caused bending of the tube and increased pressure inside the tube. These pressure changes were transmitted through the oil-filled tube and registered by the sensor. The signal was saved at a rate of 10 Hz. Data were stored in a data logger, which was attached to the side of the halter in a leather pouch. The data logger additionally recorded the 3D acceleration of head movements. Acceleration was measureable on x-, y- and z-axes. Sampling rate was set at 10 Hz (10 measurements/s) and sensitivity of the sensor at ± 10 *g*. The data logger was powered by a 260 mAh lithium-polymer battery, rechargeable via USB connection, and had an SD card, which enabled measurements over several days. It was possible to differentiate between feeding and rumination owing to the differences in the characteristics of the pressure pattern generated by each [40], [41]. MSR PC-Software [38] was used for data analysis, and the amount of time spent feeding and ruminating was calculated per 24 hour period for each cow. Head movement was assessed by summing up acceleration value changes of all three dimensions over 24 hours in each treatment, as well as calculating the variance in acceleration value changes, which served as indicator of sudden movements (e.g. displacing flies, self-grooming).

#### Nearest neighbour distance

Using the scan sampling method [42], the distance between each focal cow and the nearest (focal or non-focal) cow was recorded on the first and third experimental day outside the common resting time. Due to the size and structure of the pasture, focal cows could not all be observed at the same time but needed to be observed one after another. The observer did not interact with the cows and slowly followed the focal cow at a constant distance of 10–20 m. Each focal cow was directly observed at two times per day for 30 min between 1000 h and 1200 h and between 1330 h and 1530 h. The distance to the nearest neighbour was recorded every 5 min.

### Heart rate variability

Heart rate variability was recorded using Polar Equine (Polar Elektro Europe BV, Zug, Switzerland, 120 g), allowing a non-invasive measurement of heartbeats [32]. To improve conductivity between electrode and skin contact, electrode gel (Anandic Medical Systems AG/SA, Feuerthalen, Switzerland) was applied. A thorax belt with two integrated electrodes was fixed around the torso directly behind the forelegs. One electrode was positioned ventrally on the left side of the sternum and the other one in a distance given by the thorax belt on the left thoracic wall. A receiver for recording the data was placed between the two electrodes. The thorax belt was additionally protected by an elastic belt of about 5 cm width, and withers were additionally protected by foam material. Data were downloaded to a computer via a base station using Bluetooth (Polar Team^2^ Pro, version 1.3.0.3, Polar Electro Oy, Kempele, Finland).

All calculations were carried out using the programs Polar ProTrainer 5 Equine Edition (version 5.35.161, Polar Elektro Europe BV, Zug, Switzerland) and R version 3.1.2 [43]. The root mean square of successive differences of heartbeats (RMSSD, ms), and the ratio between RMSSD and the standard deviation of all heartbeats (SDNN, ms), RMSSD/SDNN, were calculated for 5 × 5 min time windows during 24 hours (3 × 5 min during the day [0600 h– 1059 h, 1100 h– 1559 h, 1600 h– 2059 h], 2 × 5 min during the night [2100 h– 0159 h, 0200 h– 0559 h]). To minimize influences on heartbeats caused by different levels of physical activity and to study long-term effects on heart rate variability related to the different treatments, we only considered parts of the tachogram when the animals were lying [44]. The RMSSD reflects alternations in the vago-sympathetic balance that are vagally mediated. The SDNN is a more complex parameter reflecting vagal as well as sympathetic activation. The ratio between RMSSD and SDNN is a global indicator for general changes of the vago-sympathetic balance in the organism.

Automatic correction of the tachograms was carried out using the correction routines included in the Polar Software (Polar ProTrainer 5 Equine Edition, version 5.35.161, Polar Elektro Europe BV). Data with an error rate of more than 5% were excluded from the analysis according to von Borell and colleagues [32]. The experimental design resulted in a sample size of 38 cow-days (i.e. 19 cows × 2 days) per treatment. One cow approached calving and had to be excluded from the treatment ‘functional bell’, and data collected on another cow had to be excluded from the treatment ‘silent bell’ because the strap of the bell was broken. This resulted in a remaining sample size of 37 cow-days for the treatment ‘functional bell’, 37 cow-days for ‘silent bell’ and 38 cow-days for ‘no bell’.

### Statistical analyses

Linear mixed-effects models were performed for the different outcome variables defined above using the lmer method from the lme4 package [45] in R (version 3.1.2, [43]). Statistical assumptions (normal distribution, homoscedasticity) were checked by graphical analysis of residuals. To satisfy these assumptions, the sum and variance of acceleration changes in head movements and the summed acceleration changes of leg movements needed to be log transformed.

To adequately reflect dependencies in the experimental design, the effect of observation day was nested in individual identity nested in batch. For heart rate variability, time period was additionally nested within a given observation day. In addition, a crossed random effect of calendar date was included to all models to reflect day-to-day variation that was, for example, caused by differing weather conditions and potentially affected all the simultaneously observed cows in a similar manner. The data missing due to technical problems were distributed equally over treatments and animals, and the respective sample size for each outcome variable was accounted for by the random effects.

For each outcome variable, we set up a maximum model including the fixed effects of treatment (factor with three levels: no bell, silent bell, functional bell), experimental day (factor with two levels: first and third day for lying, leg movements and nearest neighbour distance; or three levels: first, second and third day for feeding and ruminating behaviours and head movements), and their interaction. The minimal model considered was the model including treatment only. Maximum models were reduced in a step-wise backward procedure using *p* > 0.05 as the exclusion criterion. The *p*-value for the treatment is reported independently of whether or not it reaches significance. The *p*-values for the step-wise backward model selection and for treatment were calculated using a parametric bootstrap approach with 1,000 bootstrap samples as implemented in package pbkrtest; this test is more adequate than the raw likelihood-ratio test because it does not rely on large-sample asymptotic analysis and correctly takes the random-effects structure into account [46]. Post-hoc pairwise comparisons with Tukey correction were conducted using glht from the multcomp.

## Results

**Fig 3:**
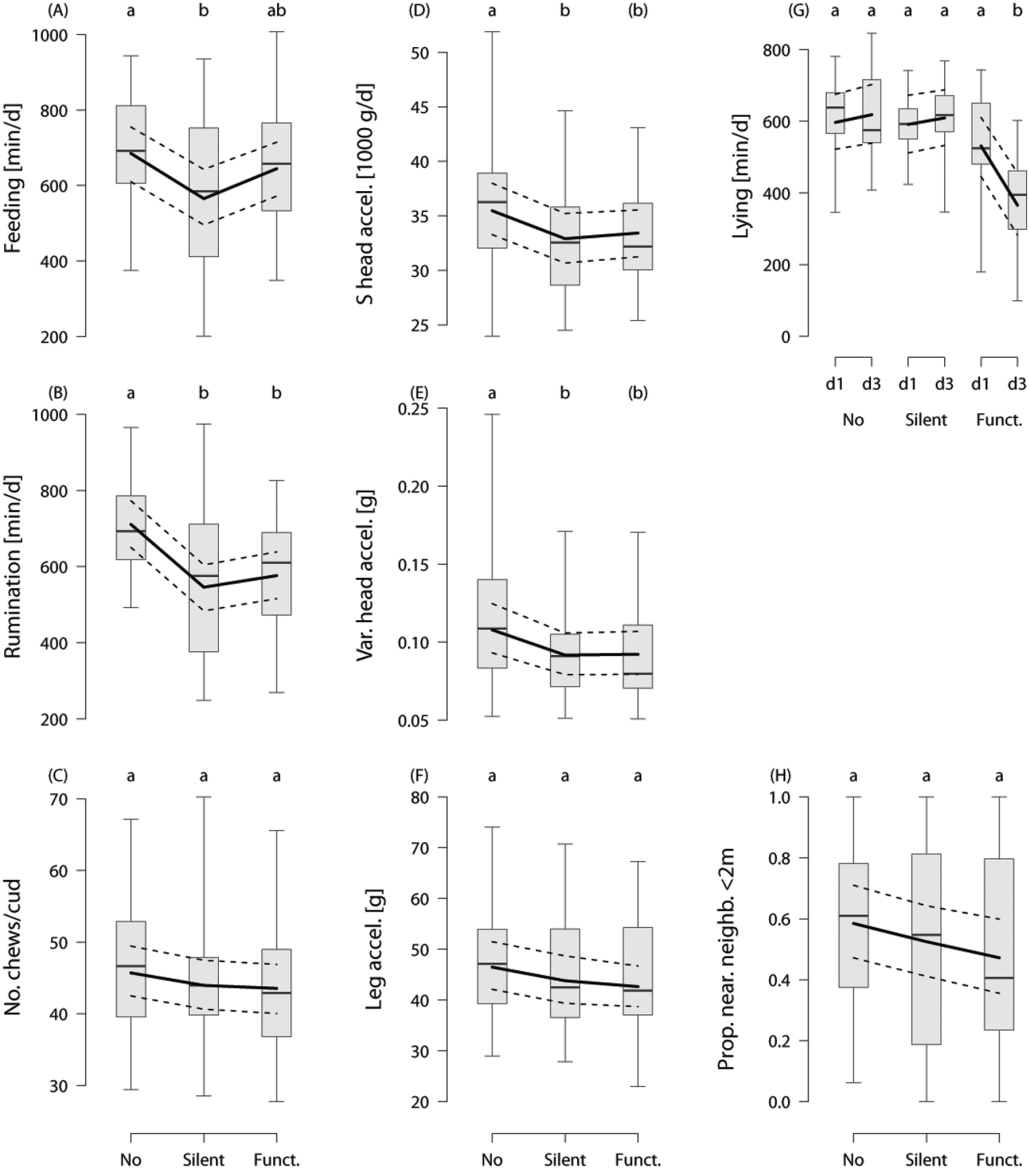
Effects of bells on the behaviour of dairy cows on pasture. A. Feeding duration per 24 hours [min], B. Duration of rumination depending on the treatment [min], C. Number of chews per rumination cud, D. Summed changes in head acceleration per 24 hours [*g*], E. Variance in head acceleration changes per 24 hours [*g*], F. Changes in leg acceleration during standing bouts [*g*] depending on the treatment, G. Lying duration per 24 hours [min] depending on the treatment and the day of observation, H. Proportion of scans with the distance to the nearest neighbour < 2 m depending on the treatment. Raw data are presented as box plots indicating observed median, first and third quartiles and absolute range of data. The solid lines show the model estimation, the dashed lines the 95% intervals of confidence. Different letters reflect significant differences (*p* ≤ 0.05) and letters in parentheses *p* < 0.1 from the Tukey corrected post-hoc comparisons.

The feeding duration with a silent bell was reduced by 115 min compared with the no bell treatment (*z* = −3.0, *p* = 0.008; Fig 3A), and by 40 min in the functional bell treatment with no significant difference to the no bell or silent bell treatment (functional bell vs. no bell: *z* = −1.0, *p* = 0.55; functional bell vs. silent bell: *z* = 1.9, *p* = 0.14). The duration of rumination was reduced by 131 min in the functional bell treatment compared with the no bell treatment (*z* = −3.1, *p* = 0.005) and by 160 min in the silent bell treatment compared with the no bell treatment (*z* = −3.8, *p* < 0.001; Fig 3B). There was no difference between the silent bell and the functional bell treatments (*z* = 0.7, *p* = 0.76). The number of chews per cud numerically decreased from the no bell to the silent bell and the functional bell treatments, but this pattern did not reach significance (Fig 3C). There was no effect of the experimental day on any of the feed intake behaviours.

The summed changes in acceleration of head movements were larger with no bell compared with the silent bell (*z* = −2.68, *p* = 0.02; Fig 3D, S2 Fig) and tended to be larger with no bell compared with the functional bell (*z* = −2.1, *p* = 0.09). There was no difference in the summed changes in head acceleration between the silent bell and the functional bell (*z* = 0.5, *p* = 0.8). The variability of head accelerations showed a similar pattern in that there was a reduction in variability with the silent bell (*z* = −2.4, *p* = 0.04; Fig 3E) and a tendency for a reduction with the functional bell (*z* = −2.3, *p* = 0.06) compared with no bell. Leg acceleration during standing bouts was slightly but not significantly reduced with the silent bell and the functional bell (Fig 3F). There was no effect of experimental day on leg movements.

The lying duration with the functional bell was reduced by 231 min on the third day of treatment compared with no bell on the first day (significant treatment x day interaction followed by post-hoc pairwise comparisons; day 3 with functional bell vs. all other day-treatment combinations: *z* ≤-4.3, *p* ≤ 0.01; all other comparisons: *z* ≥ −1.5, *p* ≥ 0.6; Fig 2G). With the functional bell, lying duration was reduced by 65 min on the third day compared with the first day (*z* ≤ −3.0, *p* = 0.04). The proportion of scans with another cow in a distance of up to 2 m to the focal animal slightly decreased from the no bell to the silent bell and the functional bell treatments, but this pattern did not reach significance (Fig 3H). There was no significant effect of treatment or experimental day on RMSSD (all *p* ≥ 0.6) or RMSSD/SDNN (all *p* > 0.5).

## Discussion

Compared with the no bell treatment, feeding duration was reduced with a silent bell. With a functional bell, however, feeding duration was intermediate between the no bell and the silent bell treatments. Compared with the no bell treatment, rumination duration was reduced with both a silent bell and a functional bell. Lying duration clearly decreased with a functional bell on the third treatment day but did not do so with a silent bell or with no bell. All other behavioural measures showed the expected tendencies, i.e. largest effect of functional bells and less effect of silent bells compared to no bell, but these effects did not reach significance. Heart rate variability was not affected by the applied treatments. There was no effect of experimental day on any of the variables except lying duration, which was reduced on the third day compared with the first day with a functional bell.

There are contradictory findings regarding the effect of noise on feeding behaviour in ungulates [47]. Caribou were found to increase time spent feeding in response to increased noise exposure [48]. By contrast, feed intake in horses [4] and goats [5] decreased when animals were exposed to acute noise. In dairy cows, immediate exposure to high-intensity noise at 105 dB, which is comparable to the bells tested in the current study, reduced feed intake and milk yield [49]. However, in the studies on the domestic species, the animals were exposed to noise only during short intervals. In the current study, cows were exposed to the sound of the bells continuously. Compared with cows wearing no bell, cows wearing a silent bell reduced their feeding duration by almost 2 hours per day whereas cows wearing a functional bell reduced their feeding duration by only 40 min. Thus, the weight of the bell seemed to affect feeding behaviour more than the sound of the bell, although it is hard to explain why feeding duration was less affected by the functional bell, i.e. weight and sound, compared with the silent bell, i.e. only weight. Longer observation periods and/or a larger sample size are needed to clarify this finding.

The rumination duration, on the other hand, was clearly reduced in both bell treatments, indicating that rumination behaviour was restricted by the weight of the bell. Reduced rumination was previously observed in cows at risk of rumen acidosis [16] and in cows after regrouping, which is widely accepted as a stressful event [50]. Although rumination can take place concurrently with standing, most rumination takes place while cows are lying [51], [52]. Thus, the reduction in lying duration might to a certain extent explain the reduction in duration of rumination. Following the argumentation of Bristow and colleagues and of Munksgaard and Simonsen that reduced rumination is an indicator of stress in cattle [17], [25], the weight of the bell may have reduced the duration of rumination by triggering some kind of stress or discomfort. Consequently, a prolonged reduction in feeding and rumination might eventually challenge the animals’ health and welfare [53], [54].

Both summed head acceleration changes and variability in head acceleration changes were reduced when the cows were wearing a bell, indicating that the cows avoided intense movements like head shaking when wearing a bell. This explanation would be supported by the correlation between head acceleration changes and amplitude that was strongest with the functional bell (the recorded amplitude was also increased in the “silent” treatments to a certain extent, due to the sensitivity of the dosimeter that recorded rubbing movements between halter and skin, S2 Fig, S1 Video). Interestingly, there was no additional reduction by the sound of the bell although this was expected when the cows had learned to avoid the chiming by reducing head movements. Cows are assumed to be capable to learn noise avoidance as shown in dairy heifers that learned to avoid tape recorded milking facility noise [9]. It thus seems that the cows did not learn how to avoid the chiming, were not able to further reduce head movements or were not affected by the chime of the bell more than by the weight of the bell.

Surprisingly, cows were lying for shorter durations when wearing a functional bell on the third day of observation compared with wearing a silent bell or no bell. The lying duration was expected to increase with functional bells compared with the other treatments, because a reduced movement may quieten the bell. In agreement with our study, the lying duration in dairy cows was found to decrease when cows were exposed to 90 dB several times per day over 3 weeks [49]. Similarly, caribou exposed to military jet aircraft noise were lying down less in response to increased noise exposure [48]. Lying down is widely accepted to be a basic requirement for the well-being of dairy cows. Heifers were found to have an inelastic demand for rest of about 12 hours per day [23], and after 3 hours of lying and feed deprivation, cows compensated for lying but not for feeding [24]. The reduced lying duration (by almost 4 hours per day on the third day of observation) in combination with the reduced feeding and rumination durations as well as the reduced head movements and the numerically reduced leg acceleration changes as found in the current study indicate that the cows spent more time standing idle. Haley and colleagues [55] concluded from their study on behaviour of dairy cows in two types of housing a decrease in lying duration combined with an increase in standing idle to be probable indicators of cow discomfort.

The focal cows in this study spent the same proportion of time in close distance (< 2 m) to other cows independent of wearing a bell or not, although the chime of the bells was reduced to levels below 80 dB only at distances > 10 m. We thus assume that the need for close spatial contact was stronger than the avoidance of the chime of the bell. Nevertheless, although these effects were not significant, the mean values for the time spent in close distance to another cow, leg acceleration changes and the number of rumination chews per cud tended to decrease when cows were wearing a bell, suggesting that the behaviour of the cows may have been restricted in this situation. By contrast, the physiological measure heart rate variability did not differ between any of the treatments. Although some studies found an increase in heart rate during noise exposure in pigs and cattle [2], [8], we focussed on heart rate variability during lying periods. Heart rate variability during periods of rest is assumed to be suitable to reflect long-term effects better than heart rate [56]. However, no effect of either of the bell treatments was found, indicating no impact of wearing a bell on vago-sympathetic balance within the duration of the study.

Finally, none of the eight behavioural measures indicated habituation by the cows although all cows had had some experience wearing a bell during one summer season as heifers. This finding contradicts those from studies on goats [5], pigs [2] and cattle [8] that used short-term noise exposures. We thus assume that the continuous exposure to sound and weight prevented habituation within the 3 days of observation. A follow-up study with a longer observation period would be necessary to find out the latency to habituate to wearing a bell. Such a study could also inform us about whether the effects represent short-term behavioural adaptions to wearing a bell or long-term changes that might challenge animal welfare.

Added effects by the functional bell in comparison with the silent bell may help distinguish the effects of weight alone from further effects of the sound of the bell. The functional bell reduced lying duration on the third day of treatment more than the silent bell, indicating that the cows may have felt more uncomfortable through the sound than the weight alone. The reduced duration of rumination with a bell, independent of sound production, may be explained by the head position of the animal during rumination. Cows usually ruminate with an upright position of the head, often while lying. The weight of the bell may result in discomfort when the cows are holding the head in this position, and thus the animals reduced the duration of rumination accordingly. By contrast, the head is always lowered for feeding, i.e. grazing, probably allowing the weight of the bell to be less burdensome. Cows do not have upper incisors, and thus jerky head movements, which make the bell chime, are required to pick a cluster of grass with the tongue. Nevertheless, feeding duration was reduced by almost 2 hours with a silent bell and 40 min with a functional bell. More research is needed to completely disentangle the effects of weight and sound.

A bell usually combines weight and sound, with heavy bells being louder than light bells. Bells need to produce sound of certain (high) amplitudes because otherwise they are useless. Thus, a cow has to cope with the combined effects of weight and sound on-farm.

## Conclusion

Wearing a bell for 3 days interfered with feeding, ruminating and lying behaviours as well as head movements of cows compared with not wearing a bell, but it did not affect heart rate variability. Cows did not habituate to the bells over the 3 days of observation. The observed behavioural changes might challenge welfare if they lasted for an extended time period, but long-term observations are necessary to quantify the effects of bells on welfare.

## Supporting Information

S1 Dataset Data on behaviour. (TXT)

S2 Dataset Data on heart rate variability. (TXT)

S1 Fig Hay rack that was used to restrain the cows. (TIF)

S2 Fig Effect of head movement on amplitude measured by a dosimeter in the halter. (DOCX)

S1 Sound File Sound file of the bells used. (WAV)

S1 Video Cow grazing with a functional bell. (MP4)
