# Supplementary figures and images for: Do Bells Affect Behaviour and Heart Rate Variability in Grazing Dairy Cows?
Source: PLoS One. 2015 Jun 25;10(6):e0131632. doi: 10.1371/journal.pone.0131632 (PMC4482024; doi:10.1371/journal.pone.0131632)

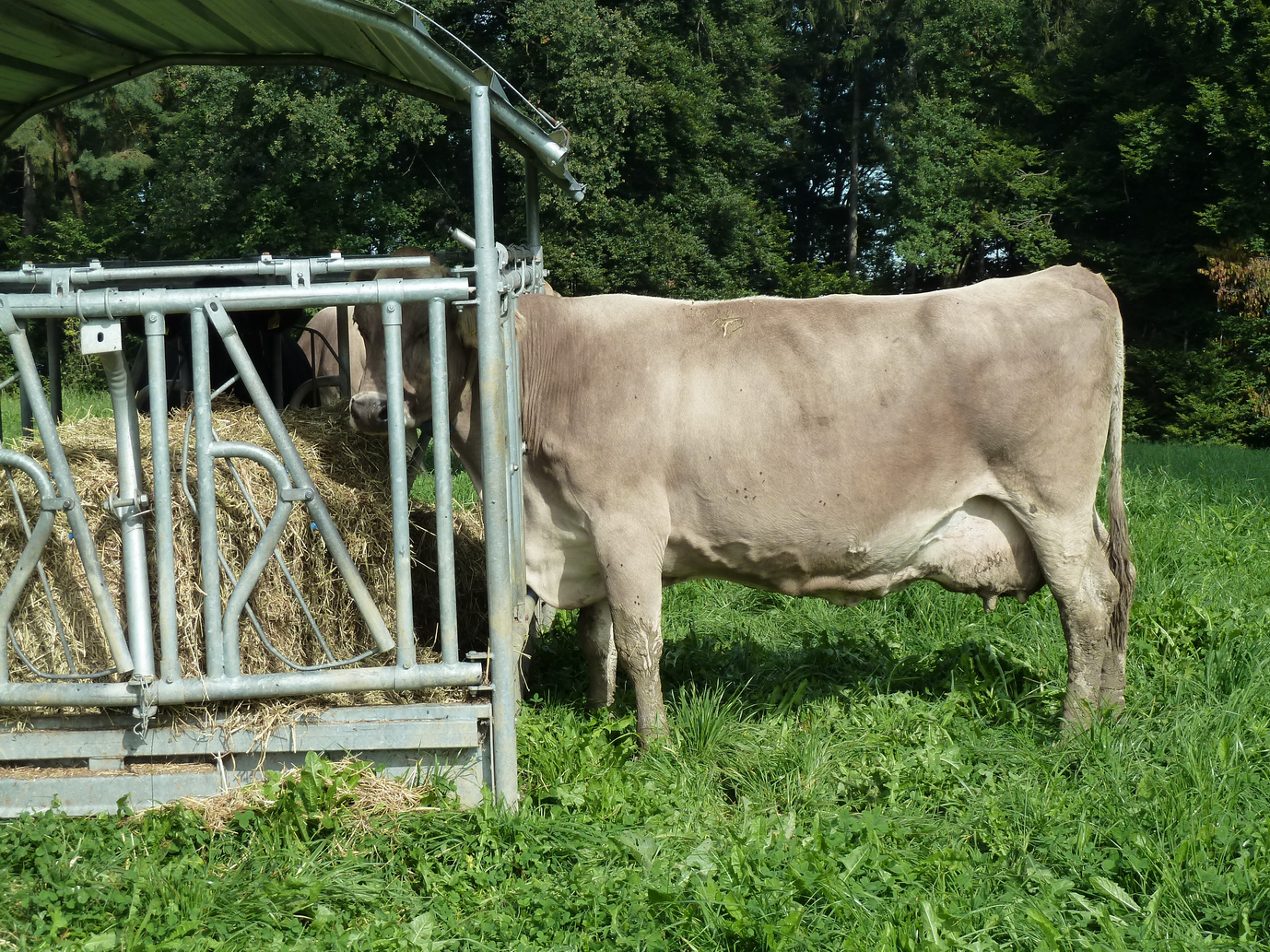

Supplement: S1 Fig — (TIF) [file pone.0131632.s003.tif]
