# Supplementary material for: Do Bells Affect Behaviour and Heart Rate Variability in Grazing Dairy Cows?
Source: PLoS One. 2015 Jun 25;10(6):e0131632. doi: 10.1371/journal.pone.0131632 (PMC4482024; doi:10.1371/journal.pone.0131632)
Supplement: S2 Fig — (DOCX) [file pone.0131632.s004.docx]

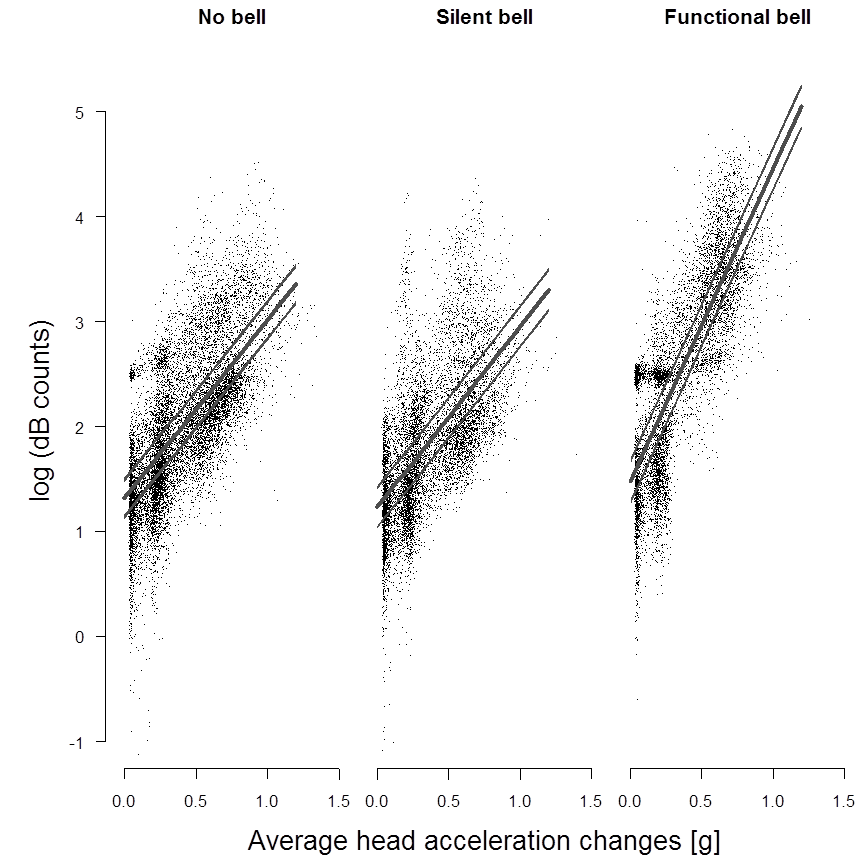


**S2 Fig.**. Effect of head acceleration changes [*g*] averaged for each available 5 min period on amplitudes of signals detected by the microphone per treatment. Points represent raw data. The thick solid lines show the model estimation for all 12 subsamples, the thin lines the 95% intervals of confidence for all 12 subsamples.

The amplitude of the sound produced by head movements (e.g. rubbing against the skin, air flow, chewing and swallowing) was recorded using the “Mini” SiSonic TM (Knowles Acoustics, LLC) a thin amplified surface mount silicon microphone built-in the data logger (MSR145) in the halter. It was hypothesized that an increase in acceleration of head movements would lead to increased amplitude of signals detected by the microphone. This effect was assumed to be strongest with a functional bell compared to both no bell and a silent bell because in addition to the sound produced by the cow itself, the chiming of the bell would lead to a clear increase in amplitude.

The acceleration changes were averaged over 5 min and combined with the counts produced by the microphone when a certain power of signal was recorded. This resulted in 576 data points for each cow in a given treatment. To reduce temporal dependency in these data, 12 random subsamples were generated and evaluated separately using the same general approach as in the other analyses. Response variable for the linear mixed effects model was the log-transformed numbers of counts. Treatment, head acceleration and their interaction were the explanatory effects. Observation day nested in cow nested in batch crossed by calendar date was used as the random effect. The *p-values* were calculated using a parametric bootstrap approach with 1,000 bootstrap samples on each of the twelve subsamples.

An increase in acceleration of head movements increased the amplitude much more with the functional bell compared with the silent bell or without bell (treatment × head acceleration changes *p*< 0.001 in all subsamples). During the direct observations we observed that walking, feeding (grazing) and rumination led to continuous chiming of the bell. These behaviours always include head movements. Running and head shaking led to a sharp increase in head movements, but these behaviours occurred rarely.
